# Drugs; Their Uses, Abuses and Measurements

**Published:** 1908-04-15

**Authors:** David Stern

**Affiliations:** Cincinnati, O.


### DRUGS; THEIR USES, ABUSES AND MEASUREMENTS.

BY DAVID STERN, B.S., D.D.S., CINCINNATI, O.

Read before the Cincinnati Odontological Society, October 25, 1907.

With the publication last fall of the last or "eighth decennial revision" of the Pharmacopeia of the United States, which became official in September, 1905, the subject of medicaments suggested itself to me as one for a paper to be read before this society at the May, 1907, meeting. It was, however, necessary to postpone the reading of this paper until this meeting, and in the meanwhile I have noticed that a paper on "Pharmacopeial Revision" was read by Dr. Edward Hoffmeister (*Cosmos*, June, 1907) before the Maryland

State Dental Association at Washington, D. C., on June 7, 1906, and also one on the same subject by Dr. G. Benton Squires (*Cosmos*, August, 1907), Somerville, Mass., before the Northeastern Dental Association at Boston on October 6, 1906. Dr. Hoffmeister's paper appeared in print in June of this year (a year after its reading), and Dr. Squires' in August of this year. They cover much of what I intended to say, but the subject is an important one and has not been discussed in this society, so we will consider the subject of drugs in regard to their uses, abuses and measurements; improvements in regard to purity, both on account of legal measures adopted by the federal government and the advancement in the scientific department of dentistry, for in treatments of diseases of the oral cavity dentistry is an applied science.

We have noticed that our eminent physicians prescribe the most simple remedies that conditions allow, and there appears to be no reason why the same principle should not apply to dental medicine. Should we review the list of drugs which we use, we would discover that we frequently use a number, any one of which would do the same work. As Dr. Kirk says: "Why first destroy the germ and its waste products with a cannon and then destroy them some more with a Gatling gun?" (*Cosmos*, November, 1905.) One reason for this mistake is that we are not sufficiently familiar with the composition of our drugs and, therefore, are not able to judge the action and the double decomposition which takes place: that is, the decomposition of the tissue and also of the drugs.

Every dental school has a cumpulsory course in chemistry and materia medica, but, to many, the former especially is considered superfluous or, perhaps, too profound, for immediately upon completing the prescribed course text books are disposed of and the subject is dismissed with a mere elementary knowledge of a very important branch, both in the operative and mechanical departments of dentistry

They forget that, having been taught the fundamental principles, the most knowledge is acquired while in practice.

We can divide drugs into two classes, as follows:

(1) Those of definite or well-known composition.

(2) Preparations of a proprietary nature, the composition of which is always problematical.

Fortunately a federal law (Pure Food and Drug Act, June 3, 1906) has been passed which in a measure controls this second class and compels the manufacturer to state, on the label, what the ingredients are, if any are of an objectionable nature, otherwise they must bear the guaranty of the commission that they are free from harmful ingredients. Like all innovations in the way of reform, methods will be found to circumvent these stringent measures, but in the course of time good results will follow. Then we will at least know what, if not how much, we are using, instead of depending upon some Greek or Latin name or the name of some deceased investigator of science. Antidolorin, Somnoform, Antikamnia, Phenalgin, Antifebrin, Sozodont, Listerine, Pasteurine, etc.. are but vague names, indicating perhaps the purposes for which these preparations are to be used, but telling nothing of what we are handling. Mothers frequently desire to quiet children while teething, but do not dare to use paregoric because it contains opium. They will, therefore, use some proprietary article in the form of a soothing syrup to put the little ones at ease, not knowing that it contains the narcotic which they desire to avoid. We will find adults using toothache chewing gum and then going to the dentist for relief from an inflamed and blistered mouth, brought on by the phenol or creosote in this gum.

We are aware of many other evils which arise from this second class of drugs, many of which give so-called relief on account of containing a large percentage of narcotics and stimulants that they are not supposed to contain. Under this Drug Act many have been compelled to change their formulas entirely and to depend upon their prior advertising for their sales. Others, who depended upon the alcoholic effect of their remedies, must now, on the labels, give the percentage of alcohol contained, and where the percentage goes beyond the legal limit they must pay the federal spirit tax, which is no small item.

After this law was enacted several months were allowed retailers to stamp their stocks on hand, so as to enable them, later on, to dispose of articles purchased prior to the enforcement of the new regulations. Not a few dealers took advantage of this and put in a large supply of such preparations, as they would be required to make the contents known if purchased after a specified date. Therefore, on general principles, we ought to ignore proprietary articles so far as possible and confine ourselves to those of the first class—articles of well-known and definite composition.

In his lecture to college students, Dr. Harlan says: "All the medicines that I use in a man's mouth are put up then and there—no patented preparations for me. If I want to be sure that a man is going to use a medicine prescribed by me, I have a boy put it up in a bottle, seal it and wrap it and give it to him, instead of sending the patient to a drug store to get the prescription filled." (*Harlan's Lectures*, page 4.) By that I presume that he has the best drugs procurable in his cabinet and does not wish to take chances with every druggist.

I will again quote the same authority: "A gentleman wishes to know what I think of Oxpara as a permanent root filling. I do not think anything about it at all, because I do not know what Oxpara is and do not use anything in the roots of teeth unless I know its composition." (Ibid.)

This is surely an example worth emulating, especially as we have so many simple and inexpensive remedies which experience has taught us to handle. To assist us in this direction, the new Pharmacopeia uses but few new synonyms in the main text, thus avoiding much confusion in prescribing and dispensing. For instance, the term Phenol is adopted to cover what were called Carbolic Acid, Phenic Acid, Phenyl Alcohol and Phenol. Carbolic Acid and Phenic were always absolute misleading misnomers, for their structural formulas show that Phenol is strictly an alcohol, consisting of a positive hydrocarbon radical and the negative hydroxyl.

"Arsenous Acid" and "Chromic Acid" have been changed to "Arsenic Trioxide" and "Chromium Trioxide." This is to conform with modern chemistry—that an anhydrid is not an acid so long as it is kept dry. Therefore, in an arsenical treatment for devitalization, the destructive acid only appears in the presence of moisture. (This is not intended as a chemical paper and these remarks on acids and oxids are merely to call attention to former mistakes in nomenclature.)

What is the advantage in having a variety of names for hydrogen peroxid, except for the manufacturers to mislead the consumers? For this preparation we now have on the market Pyrozone, Hydrogen Peroxid, Hydrogen Dioxid, Hydrozone, Dioxogen, Perhydrol, and perhaps others with whose names I am not familiar. A patient recently asked which I considered the better, Pyrozone or Dioxogen, and had to to be informed that practically there is no difference between the two; just as I had to learn that Perhydrol is the latest hydrogen peroxid preparation placed on the market.

The manufacturers claim that these names are trademarks and a guaranty of being what they represent. It appears to me that a better and less misleading plan would be for all firms to use the term hydrogen peroxid and let the name of the manufacturer on the label be an endorsement of merit. When we purchase chloroform (Squibb's) for anesthesia we are getting what the medical and dental professions consider the best. We know that nearly all of these peroxids are good, but under the Drug Act we are enabled to know their purity. For oral prophylaxis any of the commercial products will answer, but when it comes to the treatment of pathologic conditions, such as abscesses, purity is of importance and we want the real peroxid without admixtures. The last bottle of peroxid that I purchased, and it is from a reliable firm, is labeled as follows: "Hydrogen Peroxid. Each fluid ounce contains 3-16 grains of Acetanilid.

Guaranteed under the Food and Drug Act, June 30, 1906."

Acetanilid is used for preservation. H- O- loses strength with age and deterioration is accelerated by exposure to heat and light. Thus we are informed that this is not chemically pure peroxid, which could scarcely be sold to the consumer at so low a figure, for I am paying only $1.40 a gallon for the same, and it answers the purpose for which it is intended. It is a 3 per cent solution. The 30 per cent Perhydrol is guaranteed to be pure and is expensive, for this little package of 50 c. c. (about two ounces) costs $1.80, but goes a good way, as it is ten times as strong as the ordinary peroxid. The only exception I take is that it is labeled Perhydrol instead of Hydrogen Peroxid 30 per cent (Merck).

Recently I received a sample of a mouthwash labeled according to legal requirements. I will quote the formula of this article:

Alcohol 18 per cent.
Formalin $\frac{3}{4}$ min. per oz.
Acetate Amyl 1-3 min. per oz.

According to this, it takes a pint of alcohol, five pints of water, 36 drops of formalin and 12 drops of ampl acetate to make $\frac{3}{4}$ gal. of the mouth-wash, at a cost of not over 40 cents, while it is sold in a fancy bottle at 3 oz. for 25 cents. Very dilute formalin with any convenient flavoring would make just as good a mouth-wash. We should write a simple prescription for our patient, instead of recommending such proprietary articles.

Let me mention just a few additions to the Pharmacopeia, one of which is compound acetanilid powder (contains 70 per cent of acetanilid, 10 per cent caffein and 20 per cent sodium bicarbonate). Ammonal, Antikamnia, Phenalgin, Salacetin, all contain acetanilid, which the trade names endeavor to keep seceret. By prescribing this compound powder, you know just the amount of acetanilid you are giving.

Thymol Iodid (48 per cent iodin). Known by the trade names of Aristol, Annidalin, Thymotol, Cresol—a mixture of three isomeric cresols. Formerly the terms Cresol and Tricresol led to confusion.

There are other additions and changes which help us to use, as far as possible, drugs of the first class, those of definite and well-known composition, as we should. The best proof of this is that men recognized as of the greatest ability in our profession, especially in the line of materia medica, use remedies of great simplicity; men like Buckley, Black, Harlan, Rhein, Nyman, Kirk and others. They all give monopharmacy preference over polypharmacy. In modern medicine many drugs in a prescription indicate ignorance, for in such a mixture one hopes that some one of the ingredients may effect a cure. A mere chance, where failure is more liable to follow than success. Just consider the simple formulas suggested by Buckley in the treatment of putrescent pulps. (*Cosmos*, May, 1906.) In the course of treatment, eight prescriptions are given and no one has more than two ingredients; cresol and formalin, mercury bichlorid in hydrogen peroxid, potassium iodid in sarsaparilla syrup, quinine bisulphate, etc. And even the first (cresol and formalin) has been criticized, perhaps unjustly, as being a specimen of polypharmacy, on the ground that either ingredient would effect the desired result.

We know of the success in pulp extirpation which usually follows Dr. Callahan's simple sulphuric acid treatment, but this does not indicate that we must depend upon this treatment in every instance. For several years I have met with considerable success by simply filling a sensitive pulp chamber with menthol crystals, for menthol has antiseptic and anesthetic properties, putting cotton saturated with creosote over this and sealing in the same for a day. From experience and study we learn how drugs act and, after we have results, we should investigate for ourselves, for to use a remedy because an authority recommends it for a specific ailment is poor dentistry. Conditions and environment must control treatments, and these same authorities vary treatments according to existing conditions.

There is still one point, and an important one, to which not sufficient attention has been called, and that is that the metric system has been adopted in the new Pharmacopeia. This is a step in the right direction, and one that has long since been taken by nearly all foreign countries, England displaying less progress than the other European nations. England's monetary system is difficult to learn, while we can go into Germany, France, Austria or Italy and comprehend their system, which is like that of our own currency. Why should water boil at 212° in England and 100° in France? Freeze at 32° in this country and at zero in Germany? Why should a pound be of the same weight in a grocery and in a drug store, while the ounce has a different weight in the two places? Just because the apothecaries' weights have been used as standards since 1825 and it is not convenient to learn and teach the universal method. The size and weight of the grain is fixed in the mind, and because the gram is fifteen times as large and heavy it appears difficult to take it as a standard. The metric system, founded upon a decimal basis, is so simple that it can be learned from a few pages in a text book. As stated before, it makes the estimation of weights and measures as simple as the counting of American money, as compared with that of England. Weights and volumes are expressed in multiples and decimals of a universal system, of which the meter is the basis. Linear measure has the meter as the standard, and all weights and volumes can be calculated from the standard without much mental exertion. For example, on the label of one of the preparations I have mentioned this evening (peroxid), the statement that each fluid ounce contains 3-16 grains of acetanilid leaves but a vague impression of the proportion of the acetanilid to the other ingredients. In order to comprehend the percentage we must do considerable figuring.

An ounce has 480 grains.

480 grains contain 3-16 grains of acetanilid.

160 grains contain 1-16 grain of acetanilid

2,560 grains contain 1 grain of acetanilid.

1-2,560 equals 0.04 per cent of acetanilid.

Thus we see how readily we would comprehend the quantity of acetanilid if the label read:

"This preparation contains 0.04 per cent of acetanilid." With the length of the meter (about 39 inches) well impressed upon the mind, weights and volumes can easily be understood and calculated, and it is of advantage to discard all other systems and adopt the one which is used in this revised Pharmacopeia of the United States.

Although we are more or less familiar with the metric system, perhaps some present do not apply it in their practices. I have therefore brought a few of the weights and measures with me, just to show the superiority of their use over those still used by a large majority of physicians and dentists in this country.

I have also a specimen of the Perhydrol, which some of the society may not have seen.—November *Digest.*

